# Supplementary material for: A Simple, Fast, Sensitive LC-MS/MS Method to Quantify NAD(H) in Biological Samples: Plasma NAD(H) Measurement to Monitor Brain Pathophysiology
Source: Int J Mol Sci. 2024 Feb 15;25(4):2325. doi: 10.3390/ijms25042325 (PMC10888655; doi:10.3390/ijms25042325)
Supplement: Supplementary file 1 [file ijms-25-02325-s001.zip › ijms-2856125-supplementary.pdf]

Supplementary information

A Simple, Fast, and Sensitive LC-MS/MS Method for Quantification of NAD(H) in Biological Samples: Plasma NAD(H) Measurement for Monitoring Brain Pathophysiology

Tamaki Ishima [1,†], Natsuka Kimura [1,†], Mizuki Kobayashi [2], Ryozo Nagai [3], Hitoshi Osaka [2] and Kenichi Aizawa [1,4,5,*]

[1] Division of Clinical Pharmacology, Department of Pharmacology, Jichi Medical University, Shimotsuke 329-0498, Japan; ishima.tamaki@jichi.ac.jp (T.I); kimura_n@jichi.ac.jp (N.K)
[2] Department of Pediatrics, Jichi Medical University, Shimotsuke 329-0498, Japan; mizkobayashi@jichi.ac.jp (M.K); hosaka@jichi.ac.jp (H.O)
[3] Jichi Medical University, Shimotsuke 329-0498, Japan; rnagai@jichi.ac.jp (R.N.)
[4] Clinical Pharmacology Center, Jichi Medical University Hospital, Shimotsuke 329-0498, Japan
[5] Division of Translational Research, Clinical Research Center, Jichi Medical University Hospital, Shimotsuke 329-0498, Japan
* Correspondence: aizawa@jichi.ac.jp; Tel.: +81 (0)285-58-7388
† These authors contributed equally to this work.

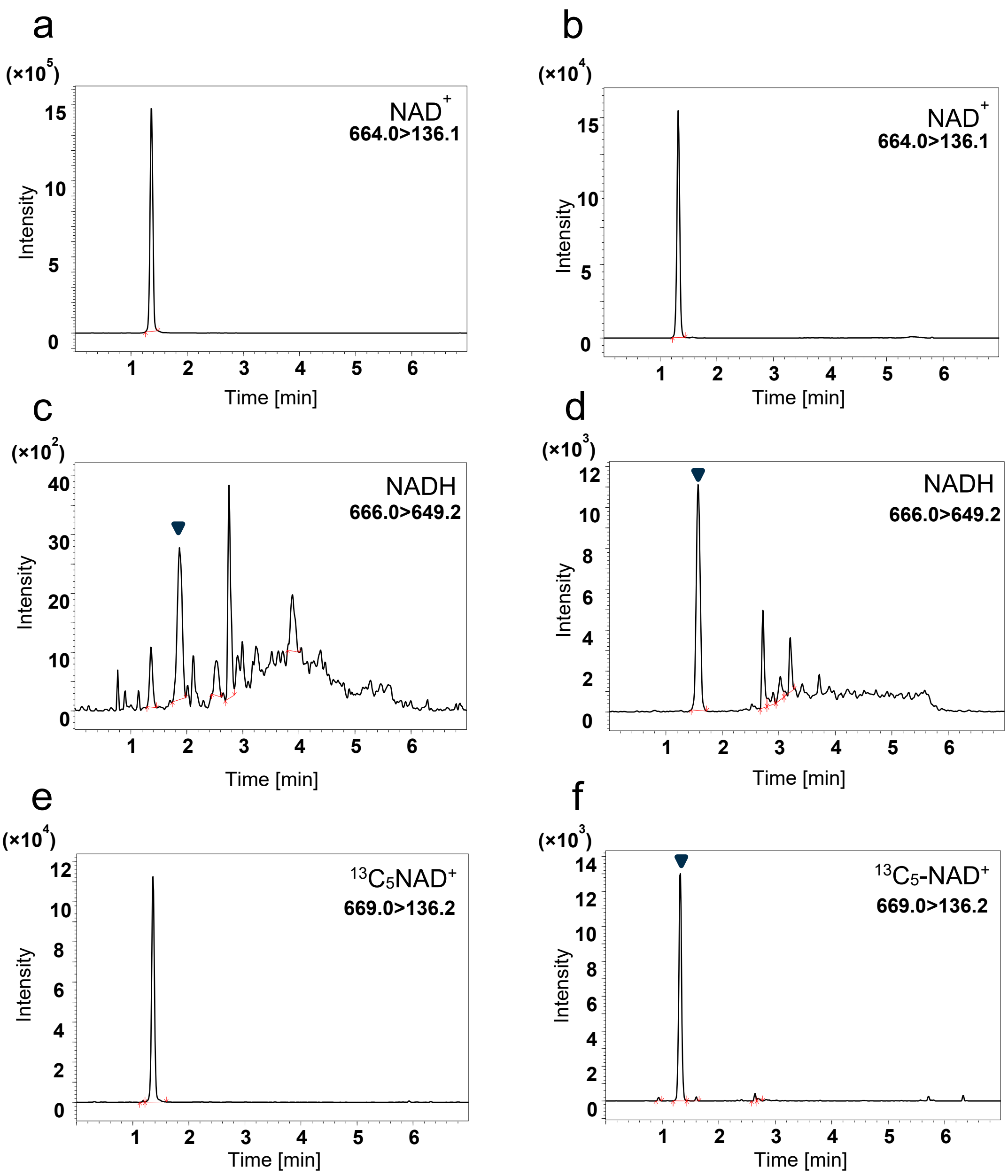

Supplementary Figure S1. Chromatograms of NAD(H) spiked in mouse whole blood and human cell matrix samples.

Chromatograms of (a) NAD$^+$ in mouse whole blood matrix, (b) NAD$^+$ in human cell matrix, (c) NADH in mouse whole blood matrix, (d) NADH in human cell matrix, (e) $^{13}C_5$- NAD$^+$ in mouse whole blood matrix, and (f) $^{13}C_5$-NAD$^+$ in human cell matrix. The stable isotope $^{13}C_5$-NAD$^+$ is the internal standard. For NAD$^+$ and NADH, the lower limits of quantification (LLOQ) for each matrix were spiked.

Supplementary Table S1. Linearity of representative calibration curves for each biological samples.

Sample	Analyte	Calibration Range	R^2	Regression equation
Mouse Brain	NAD^+	0.5-50(μM)	0.9998	y = 0.2110x +0.4844
	NADH	0.05-50(μM)	0.9996	y = 1.5856x −0.2720
Mouse Plasma	NAD^+	5-500(nM)	0.9997	y = 0.0095x +0.0488
	NADH	5-500(nM)	0.9965	y = 0.0045x −0.0640
Mouse Whole blood	NAD^+	0.5-50(μM)	0.9980	y = 0.4978x +0.3549
	NADH	0.05-50(μM)	0.9999	y = 0.2144x −0.0020
Human Cell	NAD^+	0.05-10(μM)	0.9980	y = 8.0747x +0.3993
	NADH	0.05-10(μM)	0.9994	y = 1.6762x −0.3219

Supplementary Table S2. Mean concentrations, coefficients of variation (CV), and mean accuracy of measured biological samples spiked with each analyte respective concentrations along a standard calibration curve.

Sample	Analyte	Set conc.	Mean conc.	CV (%)	Mean accuracy (%)
Mouse Brain	NAD^+	1 μM	1.23 μM	27.8	103.8
		10 μM	10.51 μM	3.9	103.0
	NADH	1 μM	0.96 μM	24.4	98.1
		10 μM	9.82 μM	7.9	98.3
Mouse Plasma	NAD^+	25 nM	27.29 nM	6.9	104.2
		250 nM	279.89 nM	2.1	110.7
	NADH	25 nM	15.00 nM	25.3	77.0
		100 nM	81.72 nM	22.4	82.2
Mouse Whole blood	NAD^+	1 μM	0.73 μM	50.3	98.2
		10 μM	11.36 μM	7.4	106.5
	NADH	1 μM	1.20 μM	16.9	115.4
		10 μM	13.03 μM	15.5	129.6
Human Cell	NAD^+	4 μM	3.88 μM	10.7	94.9
		8 μM	6.85 μM	2.8	92.0
	NADH	4 μM	2.94 μM	10.3	122.0
		8 μM	9.37 μM	4.7	105.6

Supplementary Table S3. Inter-day precision (RSD%) of this method in biological samples.

Sample	Analyte	Spiked conc.	Precision (RSD%)
Mouse Brain	NAD^+	QC low	5.2
		QC middle	5.4
		QC high	12.1
	NADH	QC low	4.6
		QC middle	10.1
		QC high	14.4
Mouse Plasma	NAD^+	QC low	8.4
		QC middle	5.0
		QC high	2.0
	NADH	QC low	6.5
		QC middle	4.9
		QC high	12.1
Mouse Whole blood	NAD^+	QC low	8.7
		QC middle	10.4
		QC high	8.1
	NADH	QC low	8.3
		QC middle	10.8
		QC high	16.2
Human Cell	NAD^+	QC low	13.0
		QC middle	6.0
		QC high	6.8
	NADH	QC low	3.8
		QC middle	16.6
		QC high	18.2

RSD: Relative standard deviation

Supplementary Table S4. Injection carryover

Sample	Analyte	ULOQ (spiked conc.)	ULOQ Area	Blank Area	LLOQ (spiked conc.)	LLOQ Area	Carryover (%)
Mouse Brain	NAD^+	50μM	24,180,277	563	0.5μM	4,389,050	0.01
	NADH	50μM	15,089,968	ND	0.05μM	179,175	-
Mouse Plasma	NAD^+	500nM	572,188	397	5nM	44,809	0.89
	NADH	500nM	248,516	ND	5nM	2,751	-
Mouse Whole blood	NAD^+	50μM	3,132,202	ND	0.5μM	6,700,189	-
	NADH	50μM	324,906	ND	0.05μM	3,988	-
Human Cell	NAD^+	10μM	7,809,828	270	0.05μM	545,266	0.05
	NADH	10μM	6,078,021	ND	0.05μM	44,204	-

ND: not detected
